# Stakeholder perspectives on implementing accreditation programs: a qualitative study of enabling factors

**DOI:** 10.1186/1472-6963-13-437

**Published:** 2013-10-24

**Authors:** Reece Hinchcliff, David Greenfield, Johanna I Westbrook, Marjorie Pawsey, Virginia Mumford, Jeffrey Braithwaite

**Affiliations:** 1 Centre for Clinical Governance Research, Australian Institute of Health Innovation, University of New South Wales, Sydney 2052, Australia; 2 Centre for Health Systems and Safety Research, Australian Institute of Health Innovation, University of New South Wales, Sydney 2052, Australia

**Keywords:** Implementation research, Accreditation, Quality improvement, Quality and safety, Health services research, Knowledge translation, Organisational context, Qualitative research, Interviews, Focus groups

## Abstract

**Background:**

Accreditation programs are complex, system-wide quality and safety interventions. Despite their international popularity, evidence of their effectiveness is weak and contradictory. This may be due to variable implementation in different contexts. However, there is limited research that informs implementation strategies. We aimed to advance knowledge in this area by identifying factors that enable effective implementation of accreditation programs across different healthcare settings.

**Methods:**

We conducted 39 focus groups and eight interviews between 2011 and 2012, involving 258 diverse healthcare stakeholders from every Australian State and Territory. Interviews were semi-structured and focused on the aims, implementation and consequences of three prominent accreditation programs in the aged, primary and acute care sectors. Data were thematically analysed to distil and categorise facilitators of effective implementation.

**Results:**

Four factors were identified as critical enablers of effective implementation: the accreditation program is collaborative, valid and uses relevant standards; accreditation is favourably received by health professionals; healthcare organisations are capable of embracing accreditation; and accreditation is appropriately aligned with other regulatory initiatives and supported by relevant incentives.

**Conclusions:**

Strategic implementation of accreditation programs should target the four factors emerging from this study, which may increase the likelihood of accreditation being implemented successfully.

## Background

Substandard healthcare is a significant global problem with serious human and financial impacts [1-3]. Accreditation programs are system-wide interventions aiming to improve the quality and safety of healthcare organisations by applying standards and promoting uptake of evidence-based clinical and organisational practices [4-6]. Despite the substantial worldwide financial investments in accreditation [7,8], the evidence-base supporting its effectiveness is weak and contradictory [6,9]. As with other quality and safety interventions [10,11], inconsistent outcomes may result from variable implementation in different settings.

Relatively little research has focused on approaches to accreditation implementation or their effectiveness. A recent review of the accreditation literature identified approximately 3,000 papers referenced in academic databases (e.g. Medline) concerning accreditation, but only 122 were classified as studies employing one or more research methods to collect or analyse data [9]. Merely 12 of these studies, in whole or in part, explicitly examined factors influencing the implementation of accreditation programs [12-23].

One study investigating program uptake and impacts amongst five Canadian healthcare organisations focused on implementation at an organisational level [23]. Distributed staff responsibility for quality and safety was identified as an important implementation facilitator. Health policies and macro-economic forces were also highlighted as critical implementation influences. In addition, health professionals were commonly found to conceptualise accreditation differently (e.g. variously as a regulatory obligation, method to obtain financial incentives, or tool to validate local quality improvement efforts), resulting in organisations enacting programs in diverse ways.

Another study investigated variables contributing to the successful implementation of accreditation programs in low and middle income countries [22]. The need for continual refinement in accreditation agency operations and program delivery was noted. The importance of two system-level factors was highlighted: ongoing and stable financial and policy support from government; and incentives for healthcare organisations to participate in programs.

These examples suggest that factors arising from program, individual, organisational and system-level domains may enable the effective implementation of accreditation programs. We conceptualise effective implementation as constituting processes that best allow accreditation programs to promote improvements to health service quality and patient safety. Yet the limited published research impedes evidence-informed design of implementation strategies. The scarcity of evidence may be due, in part, to the complexity and diversity of programs. For example, programs can involve varied combinations of activities such as: self-assessment by healthcare organisations; external assessment by surveyors on behalf of accreditation agencies; the presentation of an appraisal report to organisations; and actions undertaken by participating organisations to address recommendations [24,25]. Accreditation’s heterogeneity has largely impeded the development of implementation theories that can be generalised across different programs.

Implementation research concerning accreditation has the additional challenge of accounting for the varied and multifaceted contexts in which programs are enacted. Accreditation is practiced in a range of publicly and privately owned healthcare organisations [9]. These organisations are ‘complex adaptive systems’ [26,27] composed of, and continually modified in response to, a multitude of interconnected service delivery practices undertaken by different combinations of professionals for varied consumer groups. Furthermore, health systems respond to external pressures [28], and studies have demonstrated the influence of different macro-level factors on the implementation of complex interventions [29-31]. Due to accreditation’s role as a system-level quality and safety intervention, it is reasonable to assume that similar factors may influence the implementation of accreditation programs.

A key implication of the limited accreditation literature that addresses implementation issues is that factors enabling effective implementation are likely to be varied, arising from individual, program, organisational and system-level domains. Yet no single study has explored a wide range of factors and how they may overlap. We aimed to address this knowledge-gap by critically examining Australian healthcare stakeholders’ views regarding the range of factors influencing the implementation of three Australian accreditation programs.

## Methods

### Study context

The study forms one part of the ACCREDIT (Accreditation Collaborative for the Conduct of Research, Evaluation and Designated Investigations through Teamwork) Project, led by researchers at the Australian Institute of Health Innovation, University of New South Wales [32]. Protocols regarding current and planned ACCREDIT studies are detailed elsewhere [32-34].

The collaboration includes the leading accreditation agencies from the aged, primary and acute care sectors of the Australian health system: Aged Care Standards and Accreditation Agency (ACSAA); Australian General Practice Accreditation Limited (AGPAL); and The Australian Council on Healthcare Standards (ACHS) [35]. The specific characteristics of each agency’s accreditation program are contextually shaped by the varied sectors where they operate. For example, there is greater reliance on professional rather than peer-surveyors in the ACSAA program compared to ACHS or AGPAL. Nonetheless, these programs share the same set of core accreditation activities described previously in this paper. Therefore, the implementation of each program is likely to be mediated by a similar range of enablers.

### Sample and procedure

Management teams in the accreditation agencies provided contact details of representatives from 33 organisations they nominated as key stakeholders with significant knowledge of accreditation processes. While nation-wide representation was not explicitly sought by the research team, the representatives nominated by accreditation agencies came from multiple geographic locations (metropolitan, regional and remote) and jurisdictions (all States and Territories), providing a national perspective. The majority were drawn from acute care settings, a focus of current Australian healthcare reforms [36]. The sample was supplemented with a range of health professionals from accredited public and privately funded healthcare organisations, including medical, nursing and allied health professionals, in addition to administrative staff and managers. As accreditation has been part of the Australian health system for several decades, study participants had detailed, first-hand experience of accreditation programs and processes.

Thirty-nine focus groups and eight individual interviews involving 258 participants were conducted between August 2011 and February 2012. Focus groups involved between three and eight participants. Table 1 provides a breakdown of the data collection activities undertaken with different stakeholder groups in each healthcare sector. The research team’s prior accreditation research experience [5,21,22,24,25,37-39] suggested that thematic saturation [40] (i.e. when no new themes or categories emerge from the data) would be reached with this sample size. Focus groups were preferred for their ability to elicit communication between research participants, which helped generate additional insights from one-on-one interviews [41]. Nonetheless, eight stakeholders nominated interviews as their preferred medium of participation. Interviews and focus groups lasted approximately one hour.

**Table 1:** Study sample and data collection activities

**Stakeholder groups**	**Total number of focus groups and interviews in each health sector**			**Number of participants**
	**Acute care**	**Aged care**	**Primary care**	
Health professionals	6	3	5	80
Government health agency representatives	9	1	1	38
Health professional colleges and associations	1	4	3	41
Accreditation agency surveyors/ assessors	2	2	2	33
Accreditation agency management groups	3	2	1	51
Consumers or consumer representatives	1	1	0	15
Totals	22	13	12	258

A semi-structured interview guide was developed and used for the data collection activities. Three broad groups of issues were covered: the aims, implementation and consequences of accreditation programs. These topics were informed by prior reviews of the accreditation literature [6,9] and ongoing discussions with accreditation agency partners. Use of an open-ended guide allowed new concepts to be discussed in the focus groups and interviews. Participants were not explicitly asked to discuss factors enabling the effective implementation of programs. Instead, this information was indirectly elicited by analysing participants’ discussions of the broad issues raised. This strategy prevented the interviewers and focus group conveners from overly steering participant responses and biasing the results. Due to the widespread and long-term nature of Australian healthcare organisations’ enrolment in accreditation programs [5], implementation was conceptualised as an ongoing process rather than a discrete event. Two experienced research team members (RH and DG) collaboratively coordinated and conducted data collection.

### Recruitment

Organisations nominated as key stakeholders were recruited via email. The project and roles of participants were explained, and assurances of confidentiality provided, using information and consent forms approved by the University of New South Wales Human Research Ethics Committee [42]. Two stakeholders declined the invitation to participate in the study, producing a response rate of 94%. Health professionals were recruited at 14 educational workshops run by accreditation agencies throughout three Australian States during 2011. A convenience sample of approximately five health professionals from each workshop participated in the study.

### Analytical procedures

Two research team members (RH and DG) collaboratively reflected on their data collection experiences and discussed prominent issues raised by participants. Next, thematic analysis [43] of interview and focus group transcriptions was undertaken using textual grouping software, NVivo v.9 [44] to facilitate systematic classification of data [45]. Emergent themes were categorised into explicitly defined, overarching factors that participants perceived to enable program implementation. Analysis was performed at the individual participant level, with key findings generated using the entire dataset, rather than specific stakeholder groups. We aimed to identify factors enabling implementation that were relevant across the three accreditation programs involved in the study, as perceived by the entire range of participants. The analytical focus on developing broadly generalisable conclusions increased the likelihood of the study results being relevant to other accreditation programs operating in Australia and internationally.

The study aim was to uncover participant views and ideas, so there were no size limits placed on segments of coded text [46]. The use of a semi-structured interview guide, where the issues raised with participants were differentially focused upon and phrased to reflect their specific expertise and interests, meant that frequency counts of participant statements was not an appropriate analytical strategy. For consistency, coding was completed by one author (RH) with qualitative data analysis experience [21,47-49]. The sample was found to be sufficiently large to reach thematic saturation regarding the key study foci. The research team also aims to use additional insights drawn from the dataset, regarding accreditation programs and processes, in future studies that are not directly related to the results presented in this paper.

To increase study rigour, results were reported to Australian and international healthcare stakeholders via health services research and quality and safety conference presentations to test and confirm the validity, significance and relevance of the findings. The questions and comments received from conference participants indicated that the results were relevant and likely generalizability across different healthcare settings. The research team also discussed the results with the management teams of each accreditation agency project partner, who expressed similar support for the main study assertions.

## Results

Participant responses highlighted nine themes that collapsed into four discrete individual, program, organisational and system-level factors (Table 2). Each of the factors and themes are described in further detail below, with illustrative participant quotes.

**Table 2:** Key individual, program, organisational and system-level factors and themes perceived to enable implementation of accreditation programs

**Key factors**	**Themes**
The program is collaborative, valid and uses relevant standards	Accreditation agency use of a collaborative ethos increases healthcare organisations’ engagement in programs
	The face validity of programs is largely determined by the level of inter-survey and inter-surveyor reliability
	The clarity and focus of standards affects the perceived relevance of programs and how efficiently they can be implemented
Accreditation is favourably received by health professionals	Health professionals’ views of the benefits and costs of accreditation affects their engagement in programs
	Regular accreditation agency feedback to healthcare organisations using effective communication and framing strategies can affect professionals’ views of the value of programs
Healthcare organisations are capable of embracing accreditation	Leadership styles that champion quality and safety facilitate healthcare organisations’ uptake of CQI via accreditation
	Programs have limited capacity to drive improvements in healthcare organisations lacking cultures that support staff-wide efforts to integrate CQI into everyday practices
Accreditation is appropriately aligned with other regulatory initiatives and supported by relevant incentives	Accreditation programs are more likely to be implemented effectively when they are streamlined with other regulatory initiatives to engender a holistic approach to health system quality and safety
	Healthcare organisations’ participation in accreditation programs is encouraged by significant financial incentives that are provided by governments and insurance agencies

### The program is collaborative, valid and uses relevant standards

Participants valued accreditation agencies and programs which use a collaborative and mentoring ethos. As an accreditation agency manager noted about accreditation, *“it’s about a system that’s actually saying, ‘you know, we’re helping you do that.’ Not about saying, ‘This is where you are. When we come back next time, we’ll see if you’ve moved.’”* The ethos underlying programs was presented as critical in shaping core accreditation components.

It was commonly suggested that program implementation within healthcare organisation units is best enabled by standards focused on issues directly relevant to the daily activities of frontline health professionals. However, the challenge of developing universal standards that clearly articulate specific organisational requirements was widely acknowledged. A respondent argued, *“there’s got to be a shared understanding of what they [standards] mean … it's impossible if the organisation understands the standard to be one thing and the surveyor understands it to mean another.”*

External organisational auditing or assessment via accreditation surveys was presented as another program component contributing to effective implementation. Perceived low level inter-rater reliability within programs was seen to disengage frontline health professionals and managers. As a healthcare professional stated, *“I agree that auditing is an art and not a science, and this impacts on the validity of the survey process because you are never sure exactly what you’re going to be assessed against, which compromises the validity of the whole process.”* Several factors were viewed as capable of promoting inter-surveyor reliability, including transparent interpretation processes and surveyor workforces with appropriate capacity.

### Accreditation is favourably received by health professionals

Participants held that health professionals’ comprehension of the utility and value of accreditation modifies their engagement in programs. Professionals were characterised as often harbouring doubts about the ability of accreditation to promote organisational and health system improvements. Such views were linked to broader questions regarding the allocation of time and attention to quality improvement practices, as opposed to patient-centred clinical care, within healthcare organisations. The issue was summed up this way: *“clinicians are so busy providing care, they really don't get quality. It’s just a pain … quality is some person from some unit hidden in the bowels of the hospital … asking them to go over a whole heap of information because some people roll up with clipboards.”*

Participants opposing accreditation concentrated their critique on whether the real or even intended benefits of programs are worth the costs required for organisations to participate. A respondent expressed these concerns as follows, *“there has been no cost benefit analysis of the accreditation system and therefore it is difficult to articulate the social and economic benefits of investment … [Stakeholders] have consistently over the last decade made formal and informal comments … that the cost of accreditation is too high.”* Positive and regular accreditation agency feedback to healthcare organisations about their performance, delivered using effective communication strategies, was described as one clear way that support for accreditation amongst health professionals may be increased. There was the need, as one participant succinctly put it, *“to translate the language of accreditation to frontline staff and then for them to say, ‘oh, this is about the work we do’, and get them to understand.”* When asked to explain what effective communication of accreditation programs and requirements to health professionals may encompass, one respondent used the following terms:

“Engaging with the organisation in terms of we are here to assist and support you. This is a partnership. This is not an audit. This is not a tax investigation where we’re just going to tick boxes or cross boxes. If that partnership can develop, then there’s a real opportunity … to educate and support and strengthen and sew those seeds that the organisation can then continue to grow in the continuing cycle of improvement.”

### Healthcare organisations are capable of embracing accreditation

Two interrelated attributes of healthcare organisations were proposed to enable effective implementation of accreditation programs: credible leaders that champion continuous quality improvement (CQI) and the role of accreditation; and organisational cultures which promote collective staff ownership for CQI.

Discussions of effective leadership focused on the importance of modelling positive ideals and championing accreditation-related quality improvement practices. In describing the link between leadership and accreditation, a healthcare professional noted, *“[leadership] is what drives quality and safety because it requires people’s motivation. Accreditation is really what drives or gives us direction, but you won't get very far if you don't have that leadership and support.”* Another respondent further explained how ineffective leadership of healthcare organisations can impede the implementation of accreditation programs, *“I’ve still got leaders who don't think safety and quality should be on the agenda. So, for us, it’s [accreditation] quite difficult. And maybe that is a part of the cultural values of an organisation, but some of it is part of ingrained, you know, ageing leaders that, you know, don’t think that this is useful.”*

In addition to individual leadership, a distributed model of CQI leadership was seen as fundamentally related to the presence of organisational cultures based on collective responsibility for quality and safety. A healthcare consumer highlighted how CQI culture was seen to mediate the style of healthcare organisations’ participation in programs, *“there’s the group that just tick all the boxes for the purposes of accreditation and then there’s the group that really believe that we will do all this … because we want the improved patient care.”*

### Accreditation is appropriately aligned with other regulatory initiatives and supported by relevant incentives

Respondents reported that accreditation and other regulatory initiatives, including State or Federal licensing requirements, can be a burden on healthcare organisations. Participants emphasised the importance of accreditation programs and other regulatory initiatives coalescing to target separate, though interlinked, quality and safety priorities in healthcare delivery. A government representative interconnected them in this way: *“the basic [licensing] comes first and then the quality assurance processes and mechanisms [accreditation] build on top.”* Broad stakeholder agreement regarding the explicit role of accreditation programs, relative to other regulatory initiatives, was viewed as necessary for effective program implementation. One respondent further explained that inappropriate alignment of accreditation and regulation is a key barrier to effective quality and safety governance within their specific healthcare sector:

“The interplay between regulation and the [accreditation] standards … it’s that whole. What’s missing, from my perspective, is that whole community and industry and, you know, government, like, holistic idea of everything that we want to achieve from the system, rather than just the portions … all the different bits that we are trying to separately achieve … We’re still working at cross purposes to some extent. So the issue from my perspective is really … the global consideration of how that fits in.”

Financial incentives offered by governments and insurers to encourage organisational participation in programs were frequently highlighted. Their impact was especially emphasised by participants from the primary care sector who discussed the key role of payments from the Practice Incentives Program (PIP) [50]. One stakeholder explained it in the following terms: *“putting a financial incentive on the table gathers the swinging voters … a significant number of practices are participating because there are financial returns.”*

## Discussion

Systems-level research incorporating nation-wide perspectives is rare. To our knowledge, this is the first national study to examine stakeholders’ views on the implementation of accreditation programs. The key findings of this study are that implementation is more likely to be successful when accreditation programs and their standards are suitable and reliable, positively received by healthcare professionals and organisations, and supported by regulatory initiatives. While prior research [12-23] has identified the importance of these factors individually, this study is the first to demonstrate the importance of concurrent implementation enablers from multiple domains.

**Figure 1:**
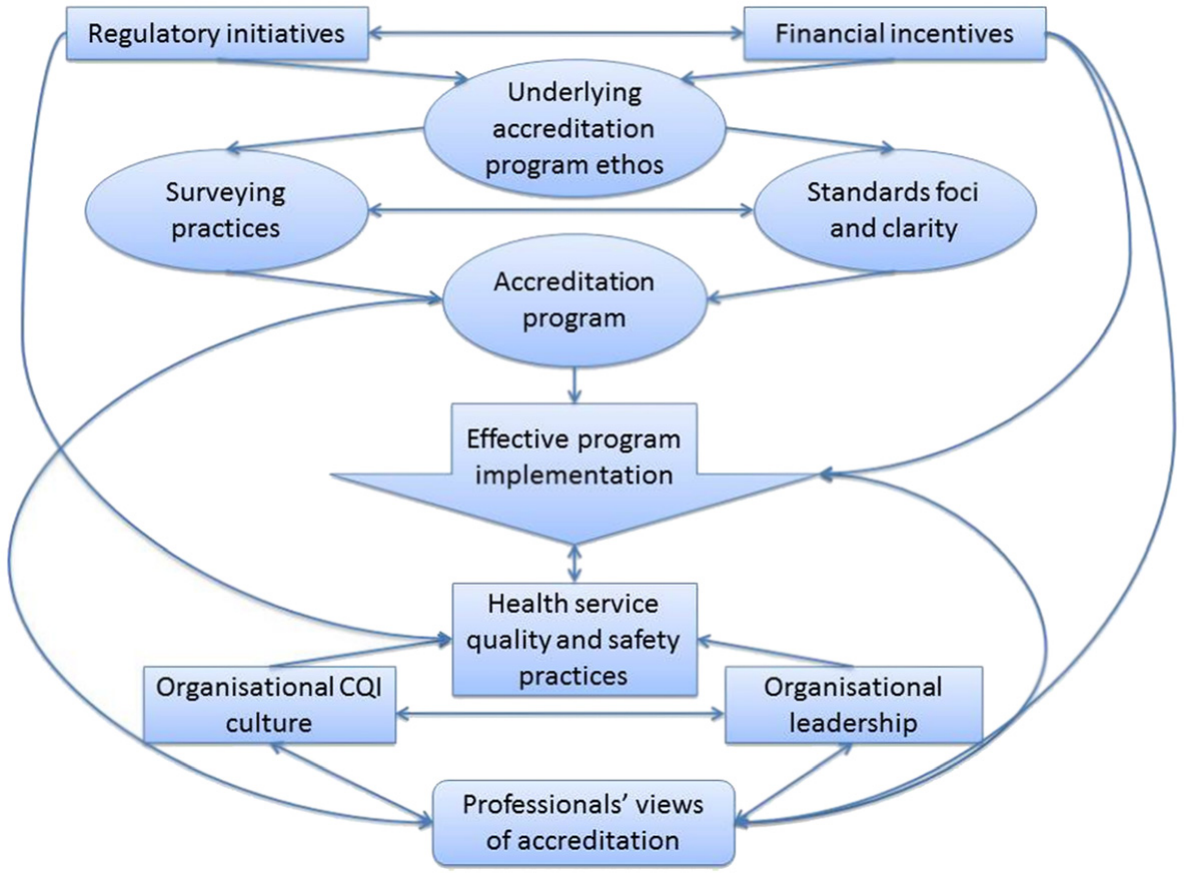
Conceptual model of the relationships between key factors and themes enabling the effective implementation of health service accreditation programs.

The conceptual model presented in Figure 1 provides a heuristic device highlighting how each of the enabling factors and themes identified in this study coalesce to facilitate effective implementation of accreditation programs. Enablers are presented in a four-tiered hierarchy spanning the systems-level, accreditation program characteristics, healthcare organisation characteristics, and the views of health professionals. Accreditation program implementation is positioned at the Figure’s centre, directly or indirectly related to each enabler. Arrows indicate the directionality of inter-enabler relationships.

The results of this study suggest that systems-level factors (that is, regulatory initiatives and financial incentives) can affect the ethos underlying accreditation programs, which affects their standards and surveying practices. Financial incentives can directly influence the views of individual health professionals regarding the benefits, relative to costs, of accreditation programs. Professionals’ views are interrelated with health service leadership and CQI culture, which are critical organisational characteristics that, along with regulatory initiatives, can influence health service quality and safety practices. The convoluted web of relationships that emerge from the results helps to draw attention to the significant challenge of implementing large-scale change in complex, adaptive healthcare systems [26,27].

Given the findings, what would help those implementing an accreditation program in Australia or internationally to increase their prospects? The findings support prior studies that highlight the influence of organisational features, including leadership and culture, on the implementation of quality and safety interventions [51,52]. Effective implementation of accreditation programs at an organisational level may require ‘change champions’ to support implementation. There may be an important role for distributed leadership [35,52] to help share experiences and institutionalise organisational cultures focused on CQI. Such cultures can foster engagement in programs, rather than pragmatic participation focused on conforming to the minimum program requirements needed to obtain financial incentives.

Participants’ views were consistent with prior research demonstrating that the components of quality and safety interventions can differentially influence their implementation [30]. However, the few evaluations of accreditation’s impacts on healthcare organisations provide limited information regarding the constituent elements of individual programs [9], such as standards and surveying methods.

The results highlight that effective implementation requires health professionals to embrace accreditation as a legitimate quality and safety tool. The literature shows that individual engagement on a mass scale can result in ‘organisational willingness to change’ [53]. Yet we uncovered that professionals’ views of the utility and value of programs were highly varied. This is problematic, as previous research has concluded that effective implementation requires divergent views of complex healthcare interventions to achieve some measure of agreement [54]. Stakeholder perceptions of accreditation were found to be influenced by the ‘framing’ [55] of programs. Effective framing necessitates use of targeted strategies to render information meaningful and engaging to diverse groups [56-58]. It follows that implementation of accreditation programs may be more effective when program aims, requirements and benefits are conceptually unified, yet articulated differently using language and formats that appeal to the cultures and normative practices of different professional groups.

Along with the presence or absence of financial incentives to promote organisational participation in accreditation, confusion regarding accreditation’s aims, compared to other regulatory mechanisms, is likely to mediate how effectively programs are implemented at a health system level. The results imply that policymakers and regulators should ensure that accreditation and other regulatory measures mutually reinforce, rather than overlap, duplicate or conflict with each other.

Our focus on three different accreditation programs operating across the Australian health system produced consistent findings that are likely to be relevant to other programs and countries. Feedback received from conference presentations [59,60] showed their congruence with international healthcare stakeholders’ experiences.

As to limitations, the sample was not randomised. Involved stakeholder groups were purposively selected by partner accreditation agencies, and healthcare professionals that participated in the study were a convenience sample. This sampling strategy may have biased the results. Mitigating this, we reached saturation of themes via a large sample of participants. Also, additional insights may have been obtained by increasing the length of time provided for focus groups. Furthermore, while rigorous methods were used to codify the views of a range of stakeholders, these remain participants’ perceptions rather than actual data on effective implementation. Additionally, while our aim was to generate broadly generalisable conclusions using the entire range of participant views, further insights could have potentially been reached by specifically comparing the views of different stakeholder groups across the three healthcare sectors involved in this study.

## Conclusions

Accreditation programs are complex quality and safety interventions, implemented adaptively and dynamically in response to varied contextual stimuli arising from different health system domains. Appreciation of the role and function of enabling factors and themes identified in this study, as well as their interrelationships, is likely to increase the likelihood of accreditation programs being implemented effectively.

## Competing interests

All authors declare that they have no competing interests.

## Authors’ contributions

JB and JIW conceived of the study. RH, DG and VM obtained the study data. All authors contributed to the interpretation and writing of the findings, and approved the final version to be published.

## Pre-publication history

The pre-publication history for this paper can be accessed here:

http://www.biomedcentral.com/1472-6963/13/437/prepub
